# Nanoemulsions and Nanostructured Lipid Carriers Containing Polyunsaturated Fatty Acids and Peptides from Mullet (*Mugil liza*)

**DOI:** 10.1021/acsomega.5c10846

**Published:** 2026-04-08

**Authors:** Camila Quadros, Karoline Pereira Rodrigues, Natália Madruga Arrieira, Diego Cabrera, Myriam Salas-Mellado, Mariano Michelon

**Affiliations:** † Laboratório de Tecnologia de Alimentos, Escola de Química e Alimentos, 67820 Universidade Federal do Rio Grande, Rio Grande 96203-900, Brasil; ‡ Laboratório de Microbiologia e Biosseparações, Escola de Química e Alimentos, Universidade Federal do Rio Grande, Rio Grande 96203-900, Brasil; § Laboratório de Análise Sensorial e Controle de Qualidade, Escola de Química e Alimentos, Universidade Federal do Rio Grande, Rio Grande 96203-900, Brasil; ∥ Centro Integrado de Análises, Universidade Federal do Rio Grande, Rio Grande 96203-900, Brasil

## Abstract

The high susceptibility of polyunsaturated fatty acids (PUFAs) to oxidation limits their application in food systems, creating the need for strategies that improve stability while preserving bioactivity. This study aimed to extract mullet (*Mugil liza*) oil by enzymatic hydrolysis, concentrate PUFAs via urea complexation, and develop lipid-based delivery systems to enhance oxidative stability and antioxidant functionality. The extracted oil contained 69% unsaturated fatty acids and remained stable for 12 months (5.91 ± 0.01 mg malondialdehyde/kg). PUFA concentration doubled the levels of eicosapentaenoic acid (EPA, 8.15 ± 0.11%) and docosahexaenoic acid (DHA, 2.64 ± 0.02%). To overcome oxidative instability, nanoemulsions (NE) and nanostructured lipid carriers (NLCs) were formulated using cocoa butter, mullet unsaturated fatty acids, Tween 80, and mullet-derived peptides, which were incorporated as natural antioxidant agents. The systems were characterized for particle size, polydispersity index (PDI), color, pH, morphology, and antioxidant capacity. NLCs exhibited particle sizes ranging from 168.3 ± 2.7 to 405.9 ± 2.8 nm and PDI values between 0.205 ± 0.06 and 0.301 ± 0.02. Formulations containing 3% peptides showed the highest ABTS^+^ (59.20–66.70%) and DPPH (28.90–38.58%) radical scavenging activities. This integrated approach, combining PUFA concentration and peptide-based nanoencapsulation from the same raw material, represents an innovative and sustainable strategy to improve stability, bioactivity, and applicability of fish-derived compounds in functional food formulations.

## Graphical abstract

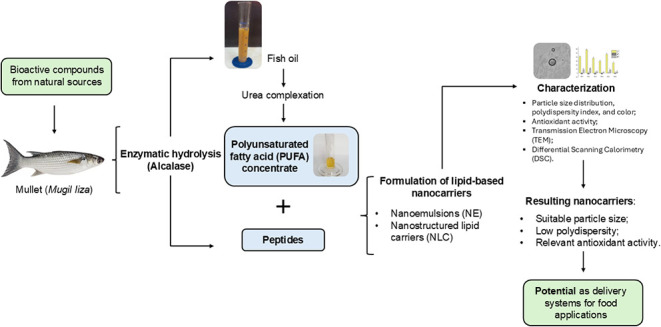

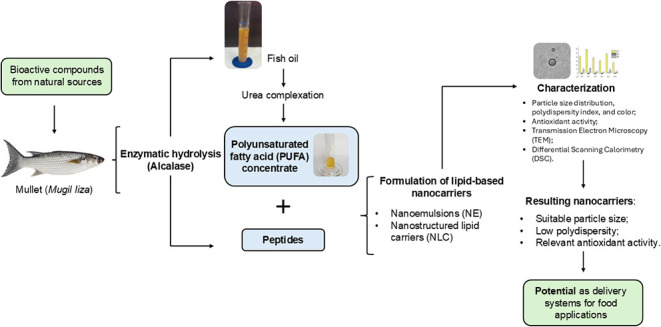

## 1 Introduction

The pursuit of a healthy diet has driven research on bioactive compounds from natural sources, considered safer alternatives to synthetic ones.^1^ In this context, fish stand out as a source of compounds with biological properties, such as peptides^2,3^ and polyunsaturated fatty acids.^4,5^ The mullet (*Mugil liza*) is a fish species rich in proteins and lipids.^3,5^ *Mugil liza* Valenciennes, 1836 is commercially relevant in Brazil, being one of the most traditional fishing resources in the Southeast and South of the country.^6^ Thus, fish stand out as a natural source of bioactive compounds, such as peptides and polyunsaturated fatty acids. The nutritional relevance of these macromolecules is closely associated with their amino acid and fatty acid compositions, which are essential parameters for assessing nutritional quality in food systems.^7,8^

Bioactive compounds can be extracted from fish through chemical or enzymatic hydrolysis reactions, or by fermentation.^9^ Among these techniques, enzymatic hydrolysis has emerged as an efficient approach for the extraction and recovery of oil from fish byproducts, providing higher lipid yields than those obtained through conventional thermal extraction methods.^10–12^

Alcalase is an enzyme produced by submerged fermentation of *Bacillus licheniformis* and has been widely employed in studies on the enzymatic hydrolysis of fish proteins.^3,11^ The resulting peptides exhibit multifunctional properties, including antimicrobial, antihypertensive, and antioxidant activities, features that have generated considerable commercial interest for the development of new products in the food, pharmaceutical, and cosmetic industries.^14^

Furthermore, enzymatic hydrolysis also represents an interesting alternative for the extraction of fish oil, which is rich in polyunsaturated fatty acids, including eicosapentaenoic acid (EPA; 20:5n-3) and docosahexaenoic acid (DHA; 22:6n-3), recognized as essential nutrients.^11,15,16^ These molecules offer a variety of health benefits, including anti-inflammatory, antioxidant, and neuroprotective properties.^16,17^ However, the high degree of unsaturation of these molecules makes them susceptible to oxidation by external factors such as exposure to air, light, metal ions, and acidic environments during food processing and storage, resulting in the loss of their health-promoting properties.^15^ Thus, strategies such as encapsulation have been widely investigated to improve the stability, bioavailability, and functionality of lipophilic bioactive compounds.^18,19^

Among food delivery systems, lipid carriers such as nanoemulsions and nanostructured lipid carriers (NLCs) stand out for increasing the surface area and facilitating the solubility and bioavailability of encapsulated compounds, in addition to being scalable processes.^18,20^ The application of nanoemulsions incorporating bioactive compounds has been shown to improve physicochemical stability and shelf life in food systems.^20,21^ Furthermore, studies have shown that the use of fish oil in systems like nanoemulsions can improve its physicochemical stability and bioavailability, expanding its potential applications.^15^ It is also noteworthy that nanostructured lipid carriers offer several advantages over other types of carriers, such as higher dispersibility of the active compound, enhanced storage stability, and improved oral bioavailability, due to their structure, which combines a mixture of different solid fats and liquid lipids.^22^ Therefore, this study aimed to develop nanoemulsions and nanostructured lipid carriers containing a concentrate of polyunsaturated fatty acids and bioactive peptides derived from mullet, evaluating their physicochemical, morphological, and bioactive properties, with a view toward application in future food formulations.

## 2 Material and Methods

### 2.1 Material

Mullet, used as the raw material for oil extraction (a source of unsaturated fatty acids) and protein hydrolyzate (a source of bioactive peptides), was obtained from the Fish Market in Rio Grande, Brazil. Organic cocoa butter used in the formulations was supplied by Sattva (Piracicaba, Brazil). The reagents ABTS (2,2′-azinobis­(3-ethylbenzothiazoline-6-sulfonic acid)) and DPPH (2,2-diphenyl-1-picrylhydrazyl) were purchased from Sigma-Aldrich. All reagents used in the preparation of lipid nanocarriers and in physicochemical analyses were of analytical grade (P.A.).

### 2.2 Peptide Production

The peptides used in this study were obtained from mullet (*Mugil liza*) byproducts through enzymatic hydrolysis using the commercial protease Alcalase, following the methodology described by Chi et al.,^23^ with modifications previously reported by Quadros et al.^3^ Briefly, the protein material was dispersed in distilled water, and the pH was adjusted to 8.0 by the addition of 1 M NaOH. The temperature was then set to 58 °C, and enzymatic hydrolysis was initiated by the addition of Alcalase at an enzyme-to-substrate ratio of 2% (w/w). The reaction was carried out under continuous agitation for 120 min. After hydrolysis, the enzyme was thermally inactivated, and the hydrolyzate was centrifuged to remove insoluble residues. The resulting supernatant, containing the peptide fraction, was collected, freeze-dried, and stored under refrigeration until further use. The peptide hydrolyzate was previously characterized in terms of degree of hydrolysis, molecular weight distribution, and antioxidant activity, as reported by Quadros et al.^3^ In the present study, these peptides were used as bioactive compounds with antioxidant potential for incorporation into nanoemulsions and nanostructured lipid carriers.

### 2.3 Oil and Fatty Acid Concentrate

#### 2.3.1 Mullet Oil Extraction

Mullet oil was obtained by enzymatic hydrolysis using Alcalase, according to Chi et al.,^23^ with modifications. Mullet portions (muscle, skin, and bones) were homogenized in distilled water (10%, w/v; protein/water) and transferred to a jacketed glass reactor (Uniglás, 190.901) connected to a thermostatic bath (Brookfield, TC-502). The reaction was conducted at pH 8.0 (adjusted with 1 M NaOH), 58 °C, and 500 rpm using a paddle stirrer (Fisatom, 715). Hydrolysis was carried out for 120 min with Alcalase (2%, w/w; enzyme/substrate). The enzyme was inactivated at 85 °C for 15 min, and the mixture centrifuged at 14,308 × g for 20 min at 4 °C. The released oil on the surface was collected and stored in amber glass bottles under refrigeration (4 °C).

#### 2.3.2 Polyunsaturated Fatty Acid (PUFA) Concentrate

The enzymatic hydrolysis of mullet oil was performed according to the method described by Gámez-Meza et al.,^24^ with modifications. Briefly, 12 mL of mullet oil was mixed with 0.05% (w/w) butylated hydroxytoluene (BHT) and 18 mL of phosphate buffer (0.1 N, pH 7.0). Lipozyme 435 was then added at a concentration of 0.5% (w/w; enzyme/oil). The reaction was carried out at 40 °C, under constant stirring (500 rpm), for 4 h and 30 min. At the end of the reaction, the mixture was centrifuged at 7,500 g for 15 min, and the hydrolyzed lipid fraction obtained was subjected to urea complexation.

Urea complexation was carried out as described by Gámez-Meza et al.^24^ The hydrolyzed oil was mixed with urea in 95% aqueous ethanol at a 1:1 (v/v) ratio. The mixture was heated and stirred until complete homogenization, then rapidly cooled by immersion in cold water and kept at −10 °C for 24 h in an ultrathermostatic bath (Quimis, M 21412, Brazil), promoting urea crystal formation. The crystals containing the complex saturated fatty acids were removed by vacuum filtration. The resulting filtrate was acidified with 1 M HCl to pH 4.0, followed by the addition of equal volumes of water preheated to 65 °C and hexane. After stirring for 1 h, the phases were separated into a separation funnel. The organic solvent was removed by evaporation, yielding PUFA concentrate.

#### 2.3.3 Differential Scanning Calorimetry (DSC)

The thermal behavior of mullet oil and PUFA concentrate was evaluated by differential scanning calorimetry (DSC) (Shimadzu, DSC-60). Approximately 5 mg of each sample was cooled to −80 °C and then heated to 80 °C at a rate of 10 °C/min.^25,26^

### 2.4 Mullet Oil Stability

#### 2.4.1 Oxidative Stability

The stability of the mullet oil was evaluated over a 12 months period at 4 °C. The sample was stored in a sealed glass vial without headspace and under light-free conditions.^27^ Lipid oxidation of the oil samples was evaluated through the quantification of thiobarbituric acid reactive substances (TBARS), according to the methodology described by Akanbi, Marshall, and Barrow.^28^ For the analysis, 100 μL of mullet oil were vortex-homogenized with 2 mL of TBARS reagent, prepared with 0.375% (w/v) thiobarbituric acid (TBA) in 250 mM HCl, for 3 min. The mixture was then centrifuged at 10,333 × g for 5 min. The lower phase was collected and heated at 80 °C for 20 min. After cooling to room temperature, the sample was centrifuged again under the same conditions. The absorbance of the bottom layer phase was measured at 535 nm using a UV/vis spectrophotometer (Kasuaki, IL 592). Quantification of TBARS values was performed using a standard curve of 1,1,3,3-tetraethoxypropane (TEP).

#### 2.4.2 Nuclear Magnetic Ressonance

The fatty acid profile of mullet oil samples over time was determined by proton nuclear magnetic resonance spectroscopy (^1^H NMR), using a Bruker High Field spectrometer (Ascend 400 MHz), operating at 9.4 T and observing ^1^H nucleus at 400.13 MHz, as described by Barison et al.^75^ For spectral acquisition, 200 μL of each sample were transferred to 5 mm NMR tubes and dissolved in 0.6 mL of CDCl_3_ (deuterated chloroform) containing 0.05% TMS (tetramethylsilane), which was used as the internal standard. The ^1^H NMR spectra were recorded at room temperature (≈20 °C), with eight accumulated scans, a relaxation delay of 1 s, a spectral width of ±9.0 ppm, and 64k data points, providing a digital resolution of 0.05 Hz. Spectral processing involved exponential multiplication of the free induction decays (FIDs) with a line-broadening factor of 0.3 Hz, followed by Fourier transformation with zero-filling up to 64k. The relaxation delay for quantitative acquisition was previously determined by inversion recovery pulse sequence experiments, with τ values ranging from 0.1 to 20 s, under the same spectral acquisition conditions. Chemical shifts were expressed in parts per million (ppm), with reference to the TMS signal set at 0.00 ppm. All pulse sequences were provided by Bruker BioSpin, and analyses were performed in triplicate. In addition, the iodine value, saponification value, and molecular weight of the samples were also determined using the ^1^H NMR technique.

### 2.5 Characterization of Mullet Oil and PUFA Concentrate

#### 2.5.1 Fatty Acid Profile by Gas Chromatography

The concentrated unsaturated fatty acids (PUFA concentrate) samples were subjected to esterification according to the method described by Metcalfe, Schmitz, and Pelka.^29^ Separation and quantification of the fatty acid methyl esters (FAMEs) were performed by gas chromatography coupled with mass spectrometry (GC–MS), using a Shimadzu 2010 Plus gas chromatograph equipped with a split/splitless injector and an RTX-1 capillary column (30 m × 0.25 mm ID × 0.25 μm film thickness). Helium was used as the carrier gas at a flow rate of 1.25 mL/min. The injector temperature was set at 260 °C, and 1 μL of sample was injected. Chromatographic separation conditions included an initial oven temperature of 50 °C, increased to 200 °C at a rate of 6 °C/min, held for 4 min. In the second heating ramp, the temperature was increased at 2 °C/min to 240 °C and held for 10 min. Fatty acids were identified by comparing retention times with methyl ester standards, and quantification was carried out by normalization of the chromatographic peak areas.

#### 2.5.2 Color

The color of mullet oil and PUFA concentrate samples was determined by colorimetry using a Minolta CR-400 colorimeter. Analyses were carried out based on the CIELAB color space parameters (L*, a*, b*).^30^

#### 2.5.3 Refractive Index

The refractive index of PUFA concentrate samples was determined at 17 °C using a digital refractometer (Hanna Instruments, model HI 96801).

### 2.6 Preparation of Lipid-Based Nanocarriers

After obtaining and characterization of mullet peptides and unsaturated fatty acids, preliminary tests (described in the Supporting Information) were conducted for the development of lipid-based nanocarriers. This step involved the nanoencapsulation of PUFA concentrate together with mullet peptides to produce two types of lipid-based delivery systems: nanoemulsions (NE) and nanostructured lipid carriers (NLC). Different combinations of solid lipid (cocoa butter), liquid lipid (mullet unsaturated fatty acids), surfactant (Tween 80), and active compound (mullet peptides) were tested to define the nanocarrier formulations, under fixed stirring conditions and varying sonication times, according to the method described by Babazadeh, Ghanbarzadeh and Hamishehkar.^22^

#### 2.6.1 Formulation of Lipid-Based Nanocarriers

Following preliminary tests, the nanoemulsion (NE) and nanostructured lipid carrier (NLC) formulations were defined, as shown in Table 1. In both systems, the dispersed phase consisted of cocoa butter and/or mullet unsaturated fatty acids, whereas the continuous phase was composed of ultrapure water and emulsifiers (Tween 80 and/or mullet-derived peptides). Different proportions of dispersed and continuous phases were evaluated, as presented in Table 1. For NLC preparation, the dispersed phase was heated to 5 °C above the melting point of cocoa butter and homogenized to obtain a clear and homogeneous dispersed phase. Subsequently, the continuous phase containing the surfactant and/or mullet-derived peptides was prepared at the cocoa butter melting temperature and added to the oil phase under continuous stirring using a rotor–stator homogenizer (IKA T10 Basic, IKA-Werke GmbH & Co. KG, Staufen, Germany) operating at 20,000 rpm for 2 min. Afterward, ultrasound treatment was applied using an ultrasonic processor (QSonica Q700, Newtown, USA) with a nominal power of 700 W and a frequency of 20 kHz, operating at 40% amplitude for 10 min.^27^

**Table 1:** Definition of Lipid Nanocarrier Formulations

Formulation	Sonication time (min)	Cocoa butter (g)	PUFA concentrate (g)	Tween 80 (g)	Peptides (g)	Water (g)
Nanoemulsions (NE)						
NE1	10	-	1.15	3.00	3.00	92.85
NE2	10	-	1.15	-	3.00	95.85
NE3	10	-	1.15	3.00	-	95.85
NE4	10	-	1.15	6.00	0.50	92.35
NE5	10	-	1.15	-	6.00	92.85
NE6	10	-	1.15	6.00	-	92.85
Nanostructured lipid carriers (NLC)						
NLC1	10	1.00	0.15	3.00	3.00	92.85
NLC2	10	1.00	0.15	-	3.00	95.85
NLC3	10	1.00	0.15	3.00	-	95.85
NLC4	10	1.00	0.15	6.00	0.50	92.35
NLC5	10	1.00	0.15	-	6.00	92.85
NLC6	10	1.00	0.15	6.00	-	92.85

### 2.7 Characterization of Lipid-Based Nanocarriers

#### 2.7.1 Particle Size Distribution, Polydispersity Index, and Color

The nanoemulsion and nanostructured lipid carrier samples containing mullet bioactive compounds were characterized in terms of particle size distribution and polydispersity index (PDI) using dynamic light scattering (DLS) with a Litesizer 500 instrument (Anton Paar). The color of the nanoemulsion and nanostructured lipid carrier samples containing mullet bioactive compounds was determined as described in Section 2.5.2.

#### 2.7.2 Transmission Electron Microscopy (TEM)

The morphology of the nanoemulsion and nanostructured lipid carrier samples containing mullet bioactive compounds was analyzed by transmission electron microscopy (TEM) using a Tecnai G2-12 Spirit Biotwin FEI microscope (Oregon, USA) operated at an acceleration voltage of 120 kV, according to Mosallaei et al.^31^ Samples were prepared by placing a drop of nanoemulsion or nanostructured lipid carrier onto a carbon-coated copper grid (200 mesh). Excess sample was removed with filter paper, and after sample adherence, a drop of 2% (w/v) uranyl acetate was added to enhance contrast. The grids were allowed to dry at room temperature and then left to rest for 24 h. Subsequently, the samples were observed by TEM.

#### 2.7.3 Differential Scanning Calorimetry (DSC)

The thermal behavior of the components of the lipid-based nanocarrier formulations (cocoa butter, peptides, and mullet unsaturated fatty acids) was also analyzed by DSC (Shimadzu, DSC-60). Approximately 3 mg of each component was cooled and then heated, using a heating rate of 2 °C/min for cocoa butter and 10 °C/min for peptides and unsaturated fatty acids.^25,26^

#### 2.7.4 Antioxidant Activity

The antioxidant activity of the mullet oil, PUFA concentrate, nanoemulsion and nanostructured lipid carrier samples containing mullet bioactive compounds was evaluated using the following methods: DPPH^•^ (2,2-diphenyl-1-picrylhydrazyl) radical scavenging and ABTS^•+^ (2,2′-azinobis­(3-ethylbenzothiazoline-6-sulfonic acid)) radical cation decolorization.

DPPH^•^ radical scavenging was determined according to the method described by Pamornpathomkul et al.^32^ Briefly, 100 μL of DPPH• solution in ethanol (200 μM) was added to 100 μL of nanoemulsion or nanostructured lipid carrier sample in a 96-well plate. The plate was then agitated and incubated at 25 °C for 30 min. Absorbance was measured at 515 nm using a Polaris microplate reader (Celer, ELISA). The DPPH^•^ solution in ethanol was used as a control. DPPH^•^ radical scavenging results were expressed as percentage inhibition.

ABTS^•+^ radical scavenging activity was determined according to the method described by Re et al.^33^ First, the ABTS^•+^ radical was generated by mixing a stock solution of ABTS (7 mM) with potassium persulfate (140 mM) and allowing the mixture to stand in the dark at room temperature for 16 h. The radical solution was then diluted with ethanol to obtain an absorbance of 0.70 ± 0.02 at 734 nm, measured using a UV/vis spectrophotometer (Kasuaki, IL 592). Subsequently, 20 μL of nanoemulsion or nanostructured lipid carrier sample was mixed with 980 μL of ABTS solution (0.1 M in 95% ethanol), vortexed, and incubated in the dark at room temperature for 6 min. Absorbance was then measured at 734 nm. ABTS^•+^ radical scavenging results were expressed as percentage inhibition.

### 2.8 Statistical Analysis

Data were evaluated in triplicate by analysis of variance (ANOVA), and means were compared using Student’s *t* test or Tukey’s test at a 5% significance level, using the software Statistica 7.0 (StatSoft, USA).

## 3 Results and Discussion

### 3.1 Extraction and Evaluation of Mullet Oil

In this study, the oil yield obtained by enzymatic hydrolysis was 38.7% relative to the total lipid content in mullet fillets, as determined by the Soxhlet method. This value may vary depending on the type of sample and the conditions applied during hydrolysis.^34^ For instance, Oliveira et al.^13^ reported a yield of 65% for oil recovery from tuna heads, whereas Lamas and Massa^10^ obtained approximately 80% for the extraction of oil from *Mustelus schmitti* livers, both using the enzyme Alcalase.

Liu, Ramakrishnan and Dave^34^ reported that the use of Alcalase results in oil of significantly higher quality, with lower oxidation and reduced free fatty acid content. Moreover, Oliveira et al.^13^ stated that enzymatic hydrolysis can be an efficient technique for oil extraction and recovery without compromising its quality, a crucial factor for its application in food or pharmaceutical products.

Table 2 presents the quality assessment parameters and the fatty acid profile of mullet oil during 12 months of storage at 4 °C. Over this period, NMR data generally showed no significant changes, except for the iodine value.

**Table 2:** Characterization of Mullet Oil during Storage at 4 °C[Table-fn tbl2fn1]

Parameter	Time (month)		
	0	1	12
Molecular weight (g/mol)	849.37 ± 12.00^a^	849.24 ± 10.44^a^	844.01 ± 10.93^a^
Iodine value (g I_2_/100 g oil)	128.96 ± 1.15^b^	128.99 ± 1.16^b^	132.97 ± 2.33^a^
Saponification value (mg KOH/g oil)	198.14 ± 2.83^a^	198.17 ± 2.46^a^	199.40 ± 2.58^a^
TBARS (mg malondialdehyde/kg oil)	0.71 ± 0.02^c^	0.84 ± 0.01^b^	5.91 ± 0.01^a^
Fatty acids profile* (%)			
Linolenic (C_18_H_30_O_2_)	13.66 ± 0.36^a^	13.77 ± 0.44^a^	14.95 ± 0.67^a^
Linoleic (C_18_H_32_O_2_)	47.60 ± 0.13^a^	47.75 ± 1.12^a^	50.32 ± 1.38^a^
Oleic (C_18_H_34_O_2_)	8.16 ± 1.00^a^	7.19 ± 0.82^a^	3.39 ± 1.09^b^
Saturated	30.58 ± 0.67^a^	31.30 ± 0.15^a^	31.20 ± 0.36^a^

i Fatty acid profile determined by NMR. Mean ± standard deviation. Different lowercase letters in the same line indicate significant difference between samples (*p* < 0.05).

The saponification value indicates the amount of KOH required to react with 1 g of oil or fat. This parameter reflects the average chain length of the fatty acids. High values suggest shorter chains and lower molecular weight.^35,36^ As shown in Table 2, saponification values remained constant during storage (198.14–199.40 mg KOH/g oil), in agreement with the stability observed in the average molecular weight of the fatty acids (849.37–844.01 g/mol). These results confirm the absence of significant changes in chain length and, consequently, in the fatty acid composition of the oil.

The iodine value is defined as the amount of iodine (in grams) that reacts with 100 g of the lipid sample. This parameter is directly related to the oil’s resistance to oxidation. High values indicate greater unsaturation; a characteristic associated with desirable technological properties.^35^ In this study, the main objective of this determination was to assess the reduction in the degree of fatty acid unsaturation due to oxidation. However, after 12 months of storage, an increase in the iodine value was observed, from 128.96 to 132 g I_2_/100 g. This atypical result is most likely associated with analytical variability or measurement error rather than actual changes in lipid composition.

In a study conducted by Hidayah et al.,^35^ oils extracted from the flesh and head of milkfish (*Chanos chanos* F.) showed iodine values of 95.29 ± 0.74 and 101.81 ± 1.46 g I_2_/100 g, respectively, which are lower than those obtained in the present study. Regarding the saponification value, the authors reported 183.90 ± 1.87 mg KOH/g for the flesh oil and 200.69 ± 1.71 mg KOH/g for the head oil, results like those observed in this study. In another study, Martinez^37^ reported an iodine value and saponification value of 146.54 ± 5.23 g I_2_/100 g oil and 186.55 ± 10.05 mg KOH/g oil, respectively, for refined mullet oil.

Ayeloja, Jimoh and Oyewole^16^ characterized oils extracted from different marine fish species (*Trachurus trachurus*, *Trachinotus blochii*, and *Scomber scombrus*) in terms of nutritional quality and fatty acid composition. The authors reported iodine values ranging from 98.53 to 118.21 g I_2_/100 g oil and saponification values between 53.29 and 89.93 mg KOH/g oil. Both parameters were lower than those observed in the present study.

Recent studies have employed ^1^H NMR to assess quality and verify label accuracy of polyunsaturated fatty acid supplements from fish oils.^38–41^ In the present study, mullet oil showed an unsaturated fatty acid content of approximately 69.0%, with linoleic acid being the predominant fatty acid, followed by linolenic and oleic acids, respectively.

Sasongko et al.^42^ evaluated the fatty acid profile of oils extracted from catfish (*Pangasius micronema* Blkr.), milkfish (*Chanos chanos* Forsskal), and snakehead (*Channa striata* Bloch.), reporting unsaturated fatty acid contents of 56.31 ± 0.01%, 50.71 ± 0.04%, and 61.55 ± 0.03%, respectively. Martinez^37^ and El-Ghafour et al.,^43^ reported 71.3% unsaturated fatty acids in whole mullet oil and 68.8% in oil extracted from mullet muscle, respectively.

Similarly, Gawad et al.^44^ investigated the lipid profile of gray mullet, identifying linoleic acid as the main polyunsaturated fatty acid, accounting for 52.4% of the total identified. These values are consistent with those observed in the present study (Table 2), which ranged from 47.60% to 50.32%. These results further highlight the potential of mullet as a promising source of unsaturated fatty acids beneficial to human health.

During the storage period, only oleic acid showed a significant decrease after 12 months, while the other fatty acids remained stable. This change may be related to the susceptibility of unsaturated fatty acids to lipid oxidation.^45,46^ Due to the high content of unsaturated fatty acids in fish oils, auto-oxidation is a natural process.^47,48^

The determination of thiobarbituric acid–reactive substances (TBARS) is an effective technique to quantify the formation of lipid oxidation products during oil degradation. The identification of these products, such as malondialdehyde (MDA), is important for assessing oxidation in oils applied in foods, as these compounds release odors, whereas primary oxidation products are odorless and tasteless.^47,48^

Boran, Karaçam and Boran,^49^ analyzed quality parameters of oils from different fish, including golden mullet, and reported a TBARS value of 9.40 mg/kg after 150 days of storage at 4 °C. The authors also indicate that the established quality and acceptability limit for human consumption is 7–8 mg malondialdehyde/kg of oil.

A study by Jia et al.^48^ investigated the oxidation of fish oil microcapsules stored for 30 days at different temperatures (4, 25, and 60 °C). The study demonstrated that the rate of TBARS formation is directly related to storage temperature, with the highest rate occurring at 60 °C (∼5 mg/kg) and the lowest at 4 °C (∼1.5 mg/kg). These results confirm that lower temperatures are an effective strategy to slow down the oxidation of fish oil.

In another study, Šimat et al.^50^ produced and characterized crude marine oils from byproducts of different fish species (farmed bluefin tuna, seabass, farmed gilthead seabream, and wild sardine). The authors observed initial TBARS values ranging from 1.2 to 2.6 μmol malondialdehyde/g oil, indicating different levels of lipid oxidation among the samples.

In the present study, TBARS results indicated good oxidative stability of the concentrated mullet oil. Initial values were low (0.71 mg/kg), with a slight increase after one month (0.84 mg/kg). Even after 12 months of storage, the value (5.91 mg/kg) remained within acceptable limits for marine oils. Korkmaz and Ozturk^27^ mentioned that TBARS values of up to approximately 5 mg malondialdehyde/kg oil have been considered acceptable for marine oils, with an emphasis on the assessment of secondary oxidation. These findings demonstrate the quality of the obtained product and its resistance to oxidation over time, highlighting its potential as a functional ingredient, provided it is stored under appropriate conditions.

### 3.2 Characterization of PUFA Concentrate

#### 3.2.1 Evaluation of Physicochemical and Thermal Properties

After the characterization of mullet oil, the concentration of unsaturated fatty acids was carried out. Table 3 presents the physicochemical and thermal properties of the oil and mullet PUFA concentrate samples, as well as the quantification of polyunsaturated fatty acids (omega-3) determined by GC–MS. The refractive index is related to the degree of saturation of the bonds and may be influenced by factors such as free fatty acid content, oxidation, and thermal treatment. In addition, it is a characteristic parameter for each type of oil.^51^

**Table 3:** Physicochemical and Thermal Properties of Mullet PUFA Concentrate[Table-fn tbl3fn1]

Parameter	Oil	PUFA concentrate
Refractive index	1.473 ± 0.01^a^	1.474 ± 0.02^a^
Color		
L*	49.61 ± 0.04^b^	61.64 ± 0.24^a^
a*	-0.13 ± 0.02^a^	-3.35 ± 0.05^b^
b*	34.70 ± 0.09^a^	31.67 ± 0.35^b^
Enthalpy (J/g)	–20.64	–8.74
Melting temperature (°C)	2.23	–18.83
Polyunsaturated fatty acids (%)		
C20:5 ω3 (EPA)	4.05 ± 0.10^b^	8.15 ± 0.11^a^
C22:6 ω3 (DHA)	1.28 ± 0.03^b^	2.64 ± 0.02^a^

i Mean ± standard deviation. Different lowercase letters in the same row indicate a significant difference between samples (*p* < 0.05).

In this study, no significant differences were observed between mullet oil and the PUFA concentrate. Similar refractive index values have been reported for oils from different fish species, such as tuna oil obtained by enzymatic hydrolysis with Alcalase (1.480),^13^ cobia oil (1.464), and cashew nut oil (1.466).^52^

The properties of oils can vary according to their fatty acid composition. Oil color is an important parameter for consumer acceptance and is related to the presence or absence of impurities. Fish oil is typically a clear liquid, ranging from yellow to brown, depending on the extraction method, whether obtained from cooking the fish or from byproduct processing.^53^ The concentration of the PUFA concentrate led to an increase in the L* parameter, indicating higher brightness/transparency, and a decrease in the a* and b* values, shifting the color toward green and reducing the intensity of yellow. These changes may have been influenced by the reduction in saturated fatty acids.

Different values were observed for the color parameters in oils from various fish species, highlighting the influence of multiple factors on their coloration. For example, in Atlantic salmon byproducts, Haq et al.^53^ reported raw oil values of L* 36.08 ± 0.66; a* 9.06 ± 0.19; b* 10.46 ± 0.17, while the PUFA concentrate showed L* 30.52 ± 0.11; a* 12.51 ± 0.38; b* 4.30 ± 0.21. In common carp, Esquerdo^54^ reported for the unsaturated fatty acid concentrate values of L* 29.76 ± 1.64; a* −1.58 ± 0.20; b* 5.14 ± 2.51. These data confirm that the color of fish oils can vary significantly depending on the species, extraction method, and degree of refinement.

Regarding the thermal properties of mullet oil and the PUFA concentrate, a high variation in melting points and enthalpy was observed, as shown in Table 3. These changes are directly related to the properties of the fatty acids present in each sample. The melting point of oils is influenced both by the degree of fatty acid unsaturation and by the carbon chain length. Therefore, oils with high iodine values and high saponification indices tend to exhibit lower melting points.^51^

As previously mentioned, Ayeloja, Jimoh and Oyewole^16^ produced oils with lower iodine and saponification values than those in the present study and reported higher melting points, ranging from 40.05 to 69.90 °C. The melting point of mullet oil is close to that of olive oil (−6 °C), while the PUFA concentrate from mullet is similar to sunflower oil (−17 °C) and soybean oil (−16 °C).^51^

From the thermograms, it can be observed that both the oil and the PUFA concentrate exhibited two pronounced peaks near −20 and 2 °C. According to Engelmann et al.,^26^ these peaks may correspond to the melting points of linoleic and oleic acids, respectively. Variations in enthalpy values may be associated with the removal of unwanted compounds, such as phospholipids and free fatty acids.

The fatty acid profiles of mullet oil and the PUFA concentrate were also determined. This analysis aimed to assess the efficiency of polyunsaturated fatty acid concentration, particularly regarding omega-3 content. Alpha-linolenic acid (ALA), docosahexaenoic acid (DHA), and eicosapentaenoic acid (EPA) are the most prominent omega-3 fatty acids. Numerous studies indicate that these compounds help reduce the risk of cardiovascular diseases and exert neuroprotective effects, aiding in the prevention of cognitive decline and neurological disorders. However, these so-called essential fatty acids are indispensable nutrients for health and must be obtained through diet.^8,55^ Furthermore, the nutritional relevance of lipids is closely associated with their fatty acid composition, which is a key parameter for assessing the nutritional quality and functional potential of food systems.^7^

As shown in Table 3 and Figure 1, the concentration of polyunsaturated fatty acids was effective, nearly doubling their levels. Thus, mullet can serve as a source of DHA and EPA for supplementation or fortifying food products.

**Figure 1:**
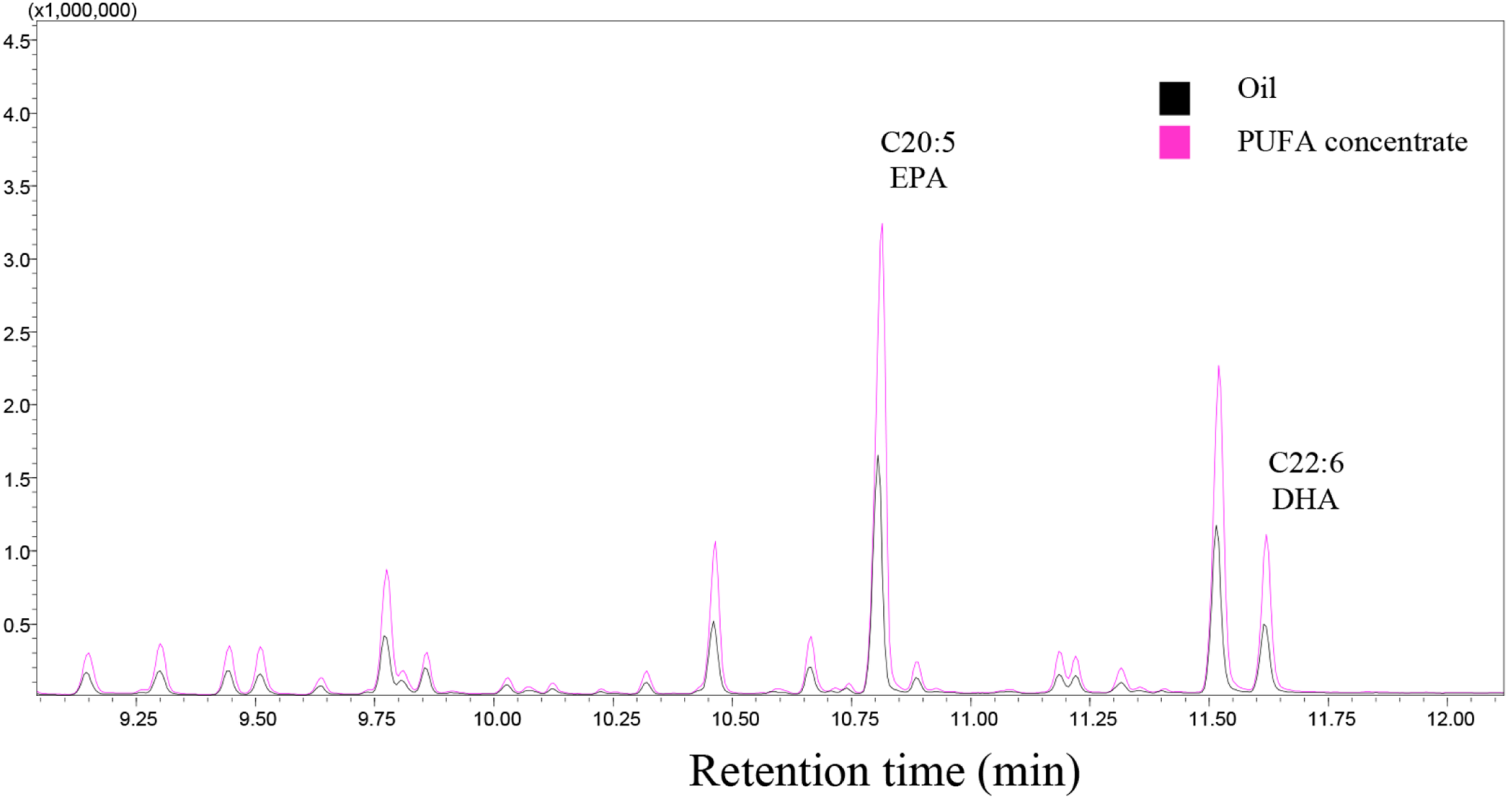
Fragment of the chromatogram of the fatty acid profile of mullet oil and PUFA concentrate.

Another mullet species (*Mugil cephalus*) has also been reported as a source of EPA (3.72%) and DHA (5.07%).^43^ Similar values to those found in this study were reported by Crexi et al.,^56^ in which PUFA concentrates obtained from carp oil (*Cyprinus carpio*) through chemical hydrolysis and urea complexation showed an increase in EPA content from 2.62% in the oil to 4.59% in the concentrate and DHA from 2.51% in the oil to 4.90% in the concentrate, representing an overall 85.4% increase in EPA + DHA content

#### 3.2.2 Antioxidant Activity

The antioxidant properties of mullet oil and the PUFA concentrate were determined using the ABTS^+^ radical scavenging and DPPH^•^ radical quenching assays, as shown in Table 4. The ABTS^+^ assay is widely used to evaluate the antioxidant activity of both hydrophilic and lipophilic compounds and is based on the reaction of hydrogen-donating antioxidant compounds with the ABTS^+^ radical.^33^ The DPPH^•^ assay, in turn, is used to assess the ability of compounds to act as free radical scavengers or hydrogen donors.^57^

**Table 4:** Antioxidant Activity of Mullet Oil and CAGI[Table-fn tbl4fn1]

Antioxidant activity	Concentration (μL/mL)	Mullet oil	PUFA concentrate
ABTS+ radical scavenging	2	13.99 ± 1.05^a^	14.12 ± 0.65^a^
	5	24.84 ± 0.65^b^	38.69 ± 1.18^a^
	10	47.97 ± 0.52^b^	55.95 ± 0.92^a^
DPPH radical scavenging	2	22.44 ± 3.85^a^	16.40 ± 0.34^b^
	5	44.97 ± 1.10^a^	41.12 ± 0.55^b^
	10	57.40 ± 2.20^a^	45.48 ± 1.35^b^

i Mean ± standard deviation. Different lowercase letters in the same row indicate significant difference between samples (*p* < 0.05).

As shown in Table 4, both mullet oil and the PUFA concentrate exhibited dose-dependent antioxidant activity in both assays. Regarding ABTS^+^ radical scavenging, the PUFA concentrate was the more effective antioxidant, achieving 55.95% activity with 10 μL of sample. However, for DPPH^•^ radical quenching, mullet oil was more efficient, reaching a maximum antioxidant capacity of 57.40% with 10 μL.

Both samples exhibited antioxidant activity, with variations in results depending on the method used. This highlights the importance of applying different assays when evaluating the antioxidant activity of various compounds. As emphasized by Ballesteros et al.,^58^ the use of multiple methods is recommended for a more comprehensive and accurate interpretation of antioxidant activity, due to the differences in the analytical principles of each assay.

### 3.3 Lipid Nanocarriers

#### 3.3.1 Preliminary Testing of NE and NLC Formulations

Recent advances in lipid nanoparticle formulation and characterization protocols highlight the critical role of surfactant choice and processing conditions in achieving stable nanostructured systems for food applications.^20^ Therefore, preliminary tests were conducted to define the optimal formulations of nanoemulsions (NE) and nanostructured lipid carriers (NLC). The first preliminary test aimed to evaluate the concentrations of the oil phase, the surfactant in the aqueous phase, and the effect of sonication on the formulation of the nanostructured lipid carriers, as shown in Table 1S Supporting Information.

Based on the results presented in Figure 1S Supporting Information, it was observed that the samples not subjected to ultrasound did not form a homogeneous phase after cooling. Additionally, the formulation with 2% oil phase showed phase separation. Therefore, a second preliminary test was conducted to evaluate the influence of sonication time on both NE and NLC formulations, as well as the effect of adding peptides as surfactants, while keeping the oil phase concentration fixed at 1.15%. The formulations studied are presented in Table 2S Supporting Information.

Based on the results obtained from the second preliminary test, the influence of sonication on the carrier formulation was evident, as proper homogenization is essential to reduce the particle size of oil droplets for encapsulation.^59^ In this study, it was found that applying 10 min of ultrasound significantly reduced the particle size compared to samples of the same formulation or those homogenized for only 2 min. Similarly, Nejadmansouri et al.^60^ reported comparable results for fish oil nanoemulsions, where the smallest particle sizes were achieved after 10 min of sonication.

As a conclusion of the second preliminary test, it was observed that NE and NLC samples showed color variations due to the addition of higher amounts of peptides. Furthermore, samples homogenized with 10 min of ultrasound were clearer, as shown in Figure 2S Supporting Information. Thus, the lipid nanocarriers were produced according to the formulations presented in Table 2 and subsequently characterized.

### 3.4 Characterization of Lipid Nanocarriers

Lipid nanocarriers are generally characterized based on parameters that primarily affect their stability and functional properties, such as particle size, polydispersity index, morphology, thermal stability, and in vitro release profile.^61^ Table 5 presents the characterization results for the nanoemulsions and nanostructured lipid carriers in terms of particle size, polydispersity index, pH, and antioxidant capacity.

**Table 5:** Characterization of Lipid Nanocarriers[Table-fn tbl5fn1]

	Diameter (nm)	PDI (−)	pH	Antioxidant capacity ABTS^+^ (%)	Antioxidant capacity DPPH^●^ (%)
Nanoemulsions					
NE1	405.9 ± 2.8^a^	0.268 ± 0.03^a^	8.07 ± 0.01^c^	65.09 ± 2.47^a^	34.83 ± 5.11^ab^
NE2	189.3 ± 3.9^c^	0.206 ± 0.02^b^	8.29 ± 0.01^b^	66.70 ± 3.09^a^	38.58 ± 4.70^a^
NE3	240.6 ± 3.6^b^	0.205 ± 0.06^b^	6.43 ± 0.01^e^	Nd	16.33 ± 1.43^d^
NE4	168.3 ± 2.7^e^	0.262 ± 0.02^ab^	6.89 ± 0.01^d^	Nd	28.32 ± 2.08^bc^
NE5	202.7 ± 5.7^c^	0.231 ± 0.02^ab^	8.46 ± 0.01^a^	27.99±0.94^b^	27.75 ± 6.95^bc^
NE6	227.8 ± 8.4^b^	0.270 ± 0.01^a^	6.84 ± 0.06^d^	Nd	24.71 ± 1.02^c^
Nanostructured lipid carriers					
NLC1	358.8 ± 10.1^a^	0.301 ± 0.02 ^a^	8.32 ± 0.01^ab^	60.34 ± 2.03^a^	28.90 ± 2.02^b^
NLC2	213.8 ± 4.2^bc^	0.240 ± 0.01^b^	8.30 ± 0.01^b^	59.20 ± 3.45^a^	32.37 ± 2.52^b^
NLC3	226.6 ± 6.4^b^	0.249 ± 0.02^ab^	6.58 ± 0.08^e^	Nd	21.10 ± 0.41^c^
NLC4	200.4 ± 7.1^c^	0.259 ± 0.01^ab^	6.79 ± 0.02^d^	Nd	20.09 ± 1.02^c^
NLC5	225.9 ± 3.9^b^	0.217 ± 0.01^b^	8.41 ± 0.01^a^	37.19 ± 2.68^b^	38.63 ± 1.64^a^
NLC6	178.6 ± 0.3^d^	0.266 ± 0.02^ab^	6.89 ± 0.01^c^	Nd	19.94 ± 3.68^c^

i NE1 (1.15% PUFA concentrate; 3% surfactant; 3% peptides); NE2 (1.15% PUFA concentrate; 3% peptides); NE3 (1.15% PUFA concentrate; 3% surfactant); NE4 (1.15% PUFA concentrate; 6% surfactant; 0.5% peptides); NE5 (1.15% PUFA concentrate; 6% peptides); NE6 (1.15% PUFA concentrate; 6% surfactant); NLC1 (0.15% PUFA concentrate; 1.0% cocoa butter; 3% surfactant; 3% peptides); NLC2 (0.15% PUFA concentrate; 1.0% cocoa butter; 3% peptides); NLC3 (0.15% PUFA concentrate; 1.0% cocoa butter; 3% surfactant); NLC4 (0.15% PUFA concentrate; 1.0% cocoa butter; 6% surfactant; 0.5% peptides); NLC5 (0.15% PUFA concentrate; 1.0% cocoa butter; 6% peptides); NLC6 (0.15% PUFA concentrate; 1.0% cocoa butter; 6% surfactant). Nd: Not detected. Mean ± standard deviation. Different lowercase letters indicate significant differences (*p* < 0.05) among samples in the same column.

#### 3.4.1 Particle Size and Polydispersity Index

Particle size influences the appearance, rheological properties, stability, and functional characteristics of lipid nanocarriers,^62^ making it one of the most important analyses in the production of delivery systems. As shown in Table 5, the formulations differed statistically from each other, with the smallest particle sizes observed in the NE4 nanoemulsion (1.15% PUFA concentrate; 6% surfactant; 0.5% peptides), followed by the NLC6 nanostructured lipid carrier (0.15% PUFA concentrate; 1.0% cocoa butter; 6% surfactant), and then the NE2 nanoemulsion (1.15% PUFA concentrate; 3% peptides); all three exhibited sizes below 200 nm.

From these results, it can be observed that cocoa butter, as well as the individual amounts of peptides and surfactant in the lipid nanocarrier formulations, did not significantly influence particle size. However, when evaluating the formulations with larger sizes, it appears that peptides and surfactant together may have formed some interfacial complex, leading to increased particle dimensions. For example, in formulations NE1 (1.15% PUFA concentrate; 3% surfactant; 3% peptides) and NLC1 (0.15% PUFA concentrate; 1.0% cocoa butter; 3% surfactant; 3% peptides), both containing 3% of each component, particle sizes exceeded 350 nm.

Fayad et al.^63^ reported that increasing the surfactant concentration (Tween 80) led to larger particle sizes in soy protein isolate nanoparticles when formulated with higher protein content, whereas in formulations with lower protein concentrations, the surfactant had the opposite effect. A similar trend was observed in this study, where the combination of peptide and surfactant concentrations resulted in increased particle sizes in both nanoemulsions and nanostructured lipid carriers (NE1 and NLC1).

The polydispersity index (PDI) is defined as an indicator of the breadth of the particle size distribution, ranging from 0 to 1.^64^ Low PDI values are associated with a more homogeneous particle size distribution.^65,66^ According to Esquerdo,^54^ higher values indicate a less uniform distribution of nanoparticle sizes; typically, diameters measured by dynamic light scattering range from 100 to 300 nm with a PDI below 0.3. In this study, the PDI of the samples ranged approximately from 0.20 to 0.30, showing varied particle sizes, similar to the results reported by Santos et al.^67^ for a fish oil nanoemulsion at 4 °C, which had a PDI of 0.22 ± 0.02. Comparable PDI values were also observed by Talesh et al.,^66^ who investigated the effect of thymol-loaded nanoemulsions (NE) and nanostructured lipid carriers (NLC) on the physiological and microbiological quality of carrots, reporting PDI values below 0.30. These findings indicate that low PDI values are characteristic of well-dispersed and physically stable lipid-based nanostructured systems, regardless of the encapsulated bioactive compound or food matrix.

#### 3.4.2 pH and Color

Regarding pH, the formulations of the two lipid systems showed significant differences, ranging from 6.43 to 8.46. This indicates that the addition of mullet peptides increased the pH of the formulations, as observed in samples NE1, NE2, NE5, NLC1, NLC2, and NLC5. According to the literature, fish oil nanoemulsions typically exhibit a pH around 5.93 ± 0.05 at 4 °C.^67^ In the present study, formulations containing only mullet oil had pH values between 6.43 and 6.89.

For the application of delivery systems, it is crucial that they do not adversely affect the product’s inherent characteristics, such as color, transparency, or turbidity, particularly when applied in beverages. Accordingly, Figure 2 presents the CIELab color space parameters for the nanoemulsions (NE) and nanostructured lipid carriers (NLC).

**Figure 2:**
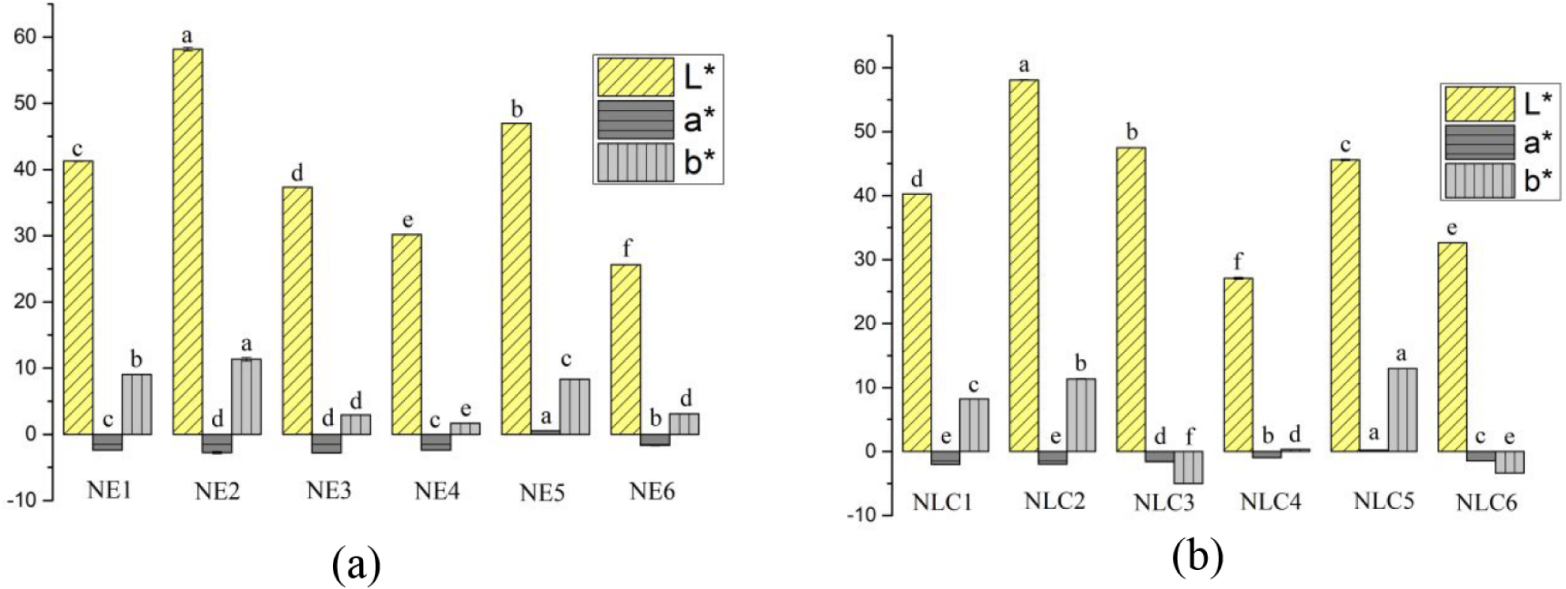
Color parameters of the (a) nanoemulsions (NE) and (b) nanostructured lipid carriers (NLC). NE1 (1.15% PUFA concentrate; 3% surfactant; 3% peptides); NE2 (1.15% PUFA concentrate; 3% peptides); NE3 (1.15% PUFA concentrate; 3% surfactant); NE4 (1.15% PUFA concentrate; 6% surfactant; 0.5% peptides); NE5 (1.15% PUFA concentrate; 6% peptides); NE6 (1.15% PUFA concentrate; 6% surfactant); NLC1 (0.15% PUFA concentrate; 1.0% cocoa butter; 3% surfactant; 3% peptides); NLC2 (0.15% PUFA concentrate; 1.0% cocoa butter; 3% peptides); NLC3 (0.15% PUFA concentrate; 1.0% cocoa butter; 3% surfactant); NLC4 (0.15% PUFA concentrate; 1.0% cocoa butter; 6% surfactant; 0.5% peptides); NLC5 (0.15% PUFA concentrate; 1.0% cocoa butter; 6% peptides); NLC6 (0.15% PUFA concentrate; 1.0% cocoa butter; 6% surfactant). Different lowercase letters indicate significant differences (*p* < 0.05) among samples for the same parameter (L, a, or b*).

It can be observed that significant variations (*p* < 0.05) occurred in the color parameters, lightness (L*) and chromas a* and b*, for the lipid nanocarrier formulations. Chroma b*, which represents the shift from blue (−) to yellow (+), showed that formulations containing peptides (NE1, NE2, NE5, NLC1, NLC2, and NLC5) had the highest b* values, followed by formulations containing mullet oil (NE3, NE4, and NE6), and finally those containing cocoa butter (NLC3, NLC4, and NLC6). Chroma a*, representing the shift from green (−) to red (+), showed no clear trend in this study. A similar result was reported by Yeşilsu and Özyurt,^59^ who studied encapsulated anchovy oil samples that exhibited lower a* values (0.22 to 0.73) and higher b* values (15.41 to 17.11).

#### 3.4.3 Antioxidant Capacity

As shown in Table 5, the nanoemulsions and nanostructured lipid carriers containing 0.5 g of peptides in their formulation (NE4 and NLC4), as well as those without peptides (NE3, NE6, NLC3, and NLC6), showed no detectable antioxidant activity in the ABTS^+^ radical scavenging assay. This result suggests that, in the formulations analyzed, the antioxidant activity is directly related to the presence of peptides.

However, the samples containing 6.0 g of peptides and no Tween 80 in their composition (NE5 and NLC5) exhibited lower antioxidant activity than those formulated with 3.0 g of peptides and Tween 80 (NE1 and NLC1), or with 3.0 g of peptides without Tween (NE2 and NLC2). These results indicate that an excess of peptides, combined with the absence of surfactant, may compromise bioactivity, possibly due to structural or stability-related interferences in the system’s dispersion.

A similar result was reported by Latorres et al.,^68^ who evaluated the antioxidant activity of white shrimp peptides nanoencapsulated in liposomes, obtaining ABTS^+^ radical scavenging values between 51.22% and 59.45%. These values are comparable to the best results obtained in the present study, suggesting that the presence of hydrogen-donating antioxidant agents, such as peptides, may explain the high activity observed.

In the DPPH^•^ radical scavenging assay, all formulations exhibited antioxidant activity. The most notable activities were observed in the formulations containing 6 and 3 g of peptides (NLC5 and NE2, respectively), highlighting the dose-dependent effect of peptide incorporation. Additionally, in peptide-free formulations, the observed antioxidant activity can be attributed to the unsaturated fatty acids from mullet oil, indicating that the developed systems can carry and preserving both hydrophilic and lipophilic antioxidant compounds.

The use of lipid nanocarriers has shown great potential in preserving the antioxidant activity of natural compounds.^69–72^ Pinto, Barros and Fonseca^72^ developed lipid nanocarrier formulations containing vegetable oil enriched with α-tocopherol. These formulations exhibited antioxidant capacities exceeding 64.3% in the DPPH^•^ radical scavenging assay. These results reinforce the effectiveness of nanocarriers in protecting and enhancing the activity of antioxidant compounds. Therefore, lipid nanocarriers represent an efficient tool for the preservation and controlled delivery of bioactive compounds, contributing to the development of formulations with high functional performance.

#### 3.4.4 Transmission Electron Microscopy (TEM)

Transmission electron microscopy (TEM) is generally applied to nanoparticles with well-defined cores and shells.^73^ Accordingly, the technique was used for samples NE1 (1.15% PUFA concentrate; 3% surfactant; 3% peptides) and NLC1 (0.15% PUFA concentrate; 1.0% cocoa butter; 3% surfactant; 3% peptides), which exhibited the largest particle sizes and highest polydispersity indices, in order to confirm the data obtained by dynamic light scattering. Figure 3 shows the images obtained for each sample.

**Figure 3:**
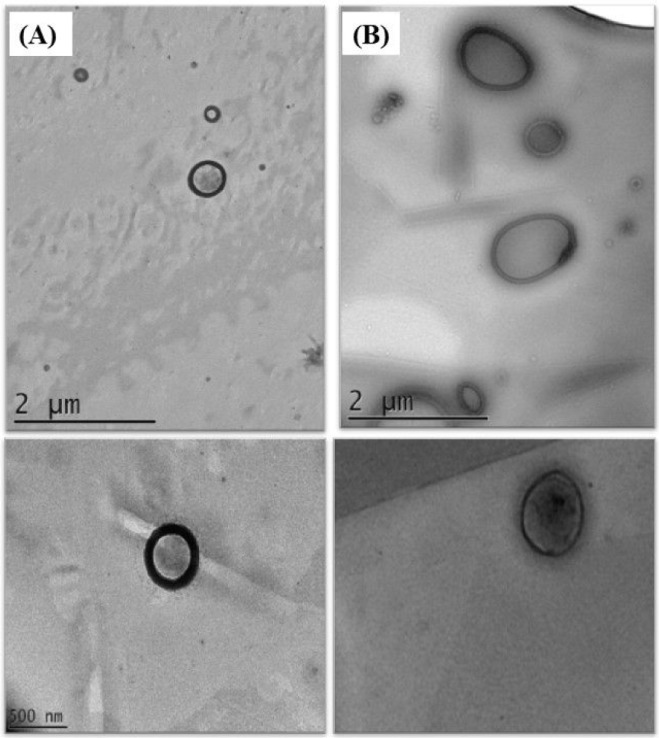
Transmission electron micrographs of the lipid nanocarriers (NE1 and NLC1): (A) Nanoemulsions; (B) Nanostructured lipid carriers.

It can be observed that the nanoemulsions exhibited a well-defined spherical shape without aggregated particles, with sizes in the nanometer range; the largest droplet measured approximately 410 nm, confirming the results from dynamic light scattering. The nanostructured lipid carriers showed similar characteristics, although their shapes varied between spherical and oval, which may have been influenced by the addition of cocoa butter.

The particles measured approximately 420 nm, a value higher than the previously determined size of around 358.8 nm using another technique. According to Chuesiang et al.,^74^ variations in particle size measurements can occur due to differences in the techniques used.

## 4 Conclusion

Bioactive compounds were successfully obtained from mullet (*Mugil liza*) through enzymatic hydrolysis, a mild and solvent-free approach. Due to its high content of unsaturated fatty acids (69%), mullet proved to be an efficient raw material for producing an omega-3–enriched oil, resulting in a 2-fold increase in EPA and DHA and improved clarity and bioactivity compared to crude oil. These results demonstrate the effectiveness of the applied strategy for concentrating health-promoting lipids from fish byproducts. To improve the oxidative stability of the PUFA concentrate and to valorize the antioxidant peptides, nanostructured lipid carriers and nanoemulsions were developed. The resulting systems exhibited suitable particle size, low polydispersity, and relevant antioxidant activity, indicating their potential as delivery systems for food applications. Future studies should focus on evaluating the long-term stability of these nanocarriers, their performance in real food matrices, and the bioavailability of the encapsulated compounds. Additionally, optimization of formulation parameters and scale-up feasibility should be investigated to support industrial application.

## Supplementary Material



## Notes

Information regarding preliminary testing for the development of nanocarriers.; tables are presented showing the conditions evaluated in the preliminary tests, such as sonication time and concentration of bioactive compounds; images of the nanocarriers formed (PDF)
