# Supplementary material for: Nanoemulsions and Nanostructured Lipid Carriers Containing Polyunsaturated Fatty Acids and Peptides from Mullet (Mugil liza)
Source: ACS Omega. 2026 Apr 8;11(15):22651–62. doi: 10.1021/acsomega.5c10846 (PMC13103841; doi:10.1021/acsomega.5c10846)
Supplement: Supplementary file 1 [file ao5c10846_si_001.pdf]

Nanoemulsions and nanostructured lipid carriers containing polyunsaturated fatty acids and peptides from mullet (*Mugil liza*)

Camila Quadros[1], Karoline Pereira Rodrigues[2], Natália Madruga Arrieira[3], Diego Cabrera[4], Myriam Salas-Mellado[1] and Mariano Michelon[2]

[1] Laboratório de Tecnologia de Alimentos, Escola de Química e Alimentos, Universidade Federal do Rio Grande, Rio Grande 96203-900, Brasil.

[2] Laboratório de Microbiologia e Biosseparações, Escola de Química e Alimentos, Universidade Federal do Rio Grande, Rio Grande 96203-900, Brasil.

[3] Laboratório de Análise Sensorial e Controle de Qualidade, Escola de Química e Alimentos, Universidade Federal do Rio Grande, Rio Grande 96203-900, Brasil.

[4] Centro Integrado de Análises, Universidade Federal do Rio Grande, Rio Grande 96203-900, Brasil.

*Corresponding author: Mariano Michelon

E-mail addresses and ORCID links.

michelonmariano@gmail.com (M. Michelon)*
https://orcid.org/0000-0003-3335-5249

camilacquadros@outlook.com (C. C. de Quadros)
https://orcid.org/0000-0001-7124-7279

karool.pereira06@gmail.com (K. P. Rodrigues)
https://orcid.org/0000-0001-5656-6802

nataliamadruga.arrieira@gmail.com (N.M. Arrieira)
https://orcid.org/0000-0002-5061-8736

diegocabrera@furg.br (D. C. Cabrera)
https://orcid.org/0000-0002-5653-1658

mysame@yahoo.com (M. M. Salas-Mellado)
https://orcid.org/0000-0002-8153-2011

SUPPLEMENTARY MATERIAL

1. PRELIMINARY TESTS FOR THE FORMULATION OF NANOEMULSIONS AND NANOSTRUCTURED LIPID CARRIERS

Table 1S. Formulation of the nanostructured lipid carrier – Test 1.

		**Oil phase**		**Aqueous phase**		
**Formulation**	**Sonication time (min)**	**Cocoa butter (g)**	**Unsaturated fatty acids (g)**	**Tween 80 (g)**	**Peptides (g)**	**Water (g)**
1	-	1.70	0.30	3.00	0.50	94.50
2	2	1.70	0.30	3.00	0.50	94.50
3	-	1.00	0.15	6.00	0.50	92.35
4	2	1.00	0.15	6.00	0.50	92.35
5	-	1.00	0.15	1.00	0.50	97.35
6	2	1.00	0.15	1.00	0.50	97.35

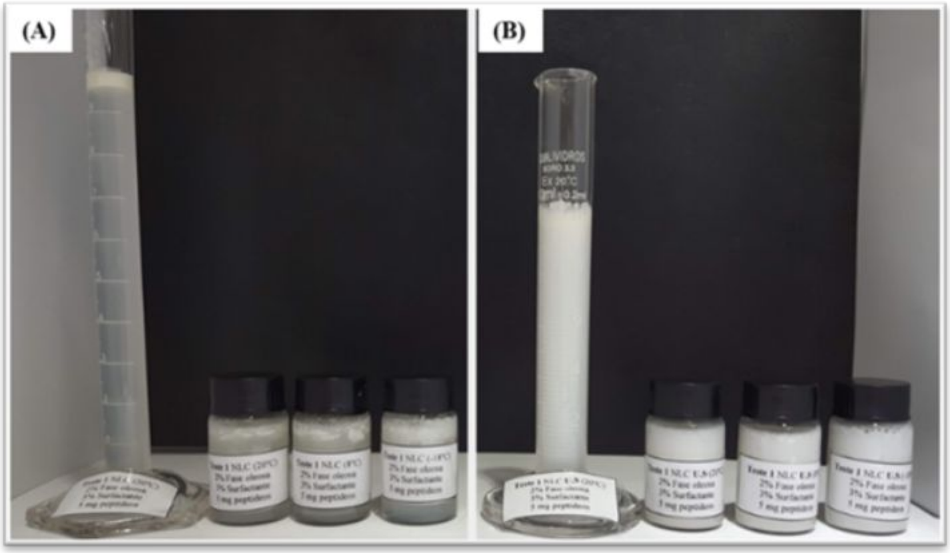

Figure S1. Nanostructured lipid carriers – Test 1. (A) Non-sonicated formulations; (B) Formulations sonicated for 2 min.

Table 2S. Formulations of nanoemulsions (NE) and nanostructured lipid carriers (NLC) – Test 2

Formulation	Sonication time (min)	Cocoa butter (g)	Unsaturated fatty acids (g)	Tween 80 (g)	Peptides (g)	Water (g)
NE1	2	-	1.15	6.00	0.50	92.35
NE1	10	-	1.15	6.00	0.50	92.35
NE2	2	-	1.15	-	6.00	92.35
NE2	10	-	1.15	-	6.00	92.35
NE3	2	-	1.15	6.00	-	92.85
NE3	10	-	1.15	6.00	-	92.85
NLC1	2	1.00	0.15	6.00	0.50	92.35
NLC1	10	1.00	0.15	6.00	0.50	92.35
NLC2	2	1.00	0.15	-	6.00	92.85
NLC2	10	1.00	0.15	-	6.00	92.85
NLC3	2	1.00	0.15	6.00	-	92.85
NLC3	10	1.00	0.15	6.00	-	92.85

Figure 2S – Lipid nanocarriers – Test 2

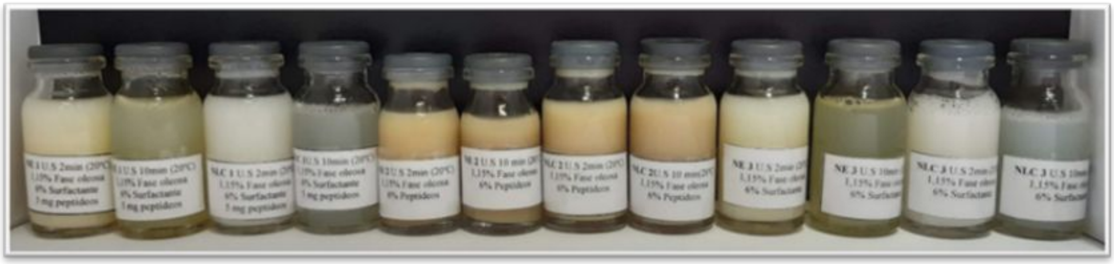

Identification of the samples, from left to right: NE1 with 2 min of ultrasonication; NE1 with 10 min of ultrasonication; NLC1 with 2 min of ultrasonication; NLC1 with 10 min of ultrasonication; NE2 with 2 min of ultrasonication; NE2 with 10 min of ultrasonication; NLC2 with 2 min of ultrasonication; NLC2 with 10 min of ultrasonication; NE3 with 2 min of ultrasonication; NE3 with 10 min of ultrasonication; NLC3 with 2 min of ultrasonication.
